# The Carbon Switch at the Level of Pyruvate and Phosphoenolpyruvate in *Sulfolobus solfataricus* P2

**DOI:** 10.3389/fmicb.2019.00757

**Published:** 2019-04-12

**Authors:** Patrick Haferkamp, Britta Tjaden, Lu Shen, Christopher Bräsen, Theresa Kouril, Bettina Siebers

**Affiliations:** ^1^Molecular Enzyme Technology and Biochemistry, Biofilm Centre, Centre for Water and Environmental Research, Faculty of Chemistry, University of Duisburg-Essen, Essen, Germany; ^2^Department of Biochemistry, University of Stellenbosch, Stellenbosch, South Africa

**Keywords:** Archaea, (hyper)thermoacidophile, *Sulfolobus solfataricus*, pyruvate kinase, phosphoenolpyruvate synthetase, carbon switch

## Abstract

*Sulfolobus solfataricus* P2 grows on different carbohydrates as well as alcohols, peptides and amino acids. Carbohydrates such as D-glucose or D-galactose are degraded via the modified, branched Entner–Doudoroff (ED) pathway whereas growth on peptides requires the Embden–Meyerhof–Parnas (EMP) pathway for gluconeogenesis. As for most hyperthermophilic Archaea an important control point is established at the level of triosephophate conversion, however, the regulation at the level of pyruvate/phosphoenolpyruvate conversion was not tackled so far. Here we describe the cloning, expression, purification and characterization of the pyruvate kinase (PK, SSO0981) and the phosphoenolpyruvate synthetase (PEPS, SSO0883) of *Sul. solfataricus*. The PK showed only catabolic activity [catalytic efficiency (PEP): 627.95 mM^-1^s^-1^, 70°C] with phosphoenolpyruvate as substrate and ADP as phosphate acceptor and was allosterically inhibited by ATP and isocitrate (*K*_i_ 0.8 mM). The PEPS was reversible, however, exhibited preferred activity in the gluconeogenic direction [catalytic efficiency (pyruvate): 1.04 mM^-1^s^-1^, 70°C] and showed some inhibition by AMP and α-ketoglutarate. The gene *SSO2829* annotated as PEPS/pyruvate:phosphate dikinase (PPDK) revealed neither PEPS nor PPDK activity. Our studies suggest that the energy charge of the cell as well as the availability of building blocks in the citric acid cycle and the carbon/nitrogen balance plays a major role in the *Sul. solfataricus* carbon switch. The comparison of regulatory features of well-studied hyperthermophilic Archaea reveals a close link and sophisticated coordination between the respective sugar kinases and the kinetic and regulatory properties of the enzymes at the level of PEP-pyruvate conversion.

## Introduction

Archaea resemble in their metabolic diversity and complexity bacteria and primitive eukaryotes. However, their metabolism is characterized by many new, unusual pathways and enzymes (Sato and Atomi, 2011; Bräsen et al., 2014; Nelson-Sathi et al., 2015; Wolf et al., 2016). Even the generic pathways of sugar degradation, such as the EMP and the ED pathway, have modifications as compared to the classical pathways (for reviews see Ronimus and Morgan, 2003; Verhees et al., 2003; Siebers and Schönheit, 2005; Bräsen et al., 2014). Most catalyzed reactions as well as the intermediates of the modified archaeal pathways resemble the classical glycolytic pathways in bacteria and eukaryotes. However, many of the utilized archaeal enzymes share no homology with their bacterial and eukaryotic counterparts but are members of different ‘new’ enzyme families [e.g., ADP/ATP-dependent hexo(gluco)kinases and ADP/ATP-dependent PFK of the ribokinase enzyme family; archaeal type class I fructose-1,6-bisphosphate aldolase of the DhnA family] (for review see Bräsen et al., 2014). This ‘acquirement’ of new catalysts is often accompanied by new regulatory properties. For example all archaeal sugar kinases characterized so far exhibit no allosteric properties and thus give rise to novel control points in the central metabolic pathways. These modified archaeal pathways therefore offer great potential for metabolic engineering and synthetic biology by the combination with classical bacterial and eukaryotic features.

The thermoacidophilic crenarchaeon *Sulfolobus solfataricus* (strain P2) grows optimally at 80°C (60 – 92°C) and pH 2 – 4 (Zillig et al., 1980) and is able to maintain an intracellular pH at around 6.5 (Moll and Schäfer, 1988). The organism uses a modification of the classical ED pathway for glucose breakdown and the reverse EMP pathway for gluconeogenesis as reported earlier (Lamble et al., 2003; Ahmed et al., 2005; Bräsen et al., 2014). The branched ED pathway is promiscuous for D-glucose and D-galactose degradation; 2-keto-3-deoxygluconate (KDG) and 2-keto-3-deoxy-6-phoshogluconate (KDPG) are the characteristic intermediates of the non-phosphorylative ED (npED) and spED pathway, respectively. After aldolase cleavage pyruvate and glyceraldehyde 3-phosphate (GAP, spED) or 2-phosphoglycerate (2PG, npED) are formed and both are channeled into the lower EMP pathway forming a second molecule of pyruvate (Ahmed et al., 2005). From a genetic approach in *Sul. solfataricus* (KDG kinase knock-out) combined with metabolomics and enzymatic studies there is evidence that the spED pathway plays a major anabolic role for the generation of hexose phosphates under glycolytic growth conditions (Kouril et al., 2013b). In addition, the upper catabolic part of the EMP pathway is active (from glucose to fructose 6-phosphate) and provides hexose phosphates via hexokinase and phosphoglucose isomerase in *Sulfolobus* spp. (Nishimasu et al., 2006). Fructose-6-phosphate constitutes the precursor for pentose generation via the reverse ribulose monophosphate pathway (Orita et al., 2006), and G6P is the building block for glycogen and trehalose formation in *Sulfolobus* (König et al., 1982; Maruta et al., 1996; Kouril et al., 2008; Zaparty and Siebers, 2011). For a functional glycolysis via the EMP pathway only the PFK seems to be missing and no functional, catabolic fructose-1,6-bisphosphate aldolase (FBPA) was identified so far. Pyruvate as central metabolite is channeled into the citric acid cycle and is completely oxidized to CO_2_. Reducing equivalents are transferred into the branched respiratory chain with oxygen as terminal acceptor for energy conversion via electron transfer phosphorylation. In addition, *Sul. solfataricus* grows on various non-saccharolytic substrates such as peptides, amino acids or alcohols and the reversed EMP pathway is used for gluconeogenesis.

Thus, like in other organism pyruvate is one central hub in the metabolism of *Sul. solfataricus* that channels carbon into different anabolic and catabolic pathways implying an important metabolic control point. In the lower common part of the classical EMP pathway in Bacteria, the conversion between phosphoenolpyruvate (PEP) and pyruvate via the antagonistic enzyme couple, catabolic PK and anabolic PEPS, serves as a switch between glycolysis and gluconeogenesis. In few species in addition a PPDK is found, which catalyzes the reversible conversion of PEP and pyruvate (Evans and Wood, 1968) and in contrast to PEPS, requires P_i_ in the anabolic and PP_i_ in the catabolic direction for activity (see Equations 1–3, ΔG°’-values were calculated via eQuilibrator^1^, Flamholz et al., 2012).

(1) Pyruvate kinase (PK, EC 2.7.1.40)
       PEP + ADP → Pyruvate + ATP (ΔG°’ = -27.7 kJ/mol, -32 kJ/mol at 75°C)
(2) Phosphoenolpyruvate synthetase (PEPS, EC 2.7.9.2)
       Pyruvate + ATP + H_2_O → PEP + AMP + P_i_ (ΔG°’ = 3.8 kJ/mol, 4.5 kJ/mol at 75°C)
(3) Pyruvate:phosphate dikinase (PPDK, EC 2.7.9.1)
       Pyruvate + ATP + P_i_ ↔ PEP + AMP + PP_i_ (ΔG°’ = 19.6 kJ/mol, 23 kJ/mol at 75°C)

Till now, little is known about the PEP-pyruvate conversion and its regulatory function for the glycolytic/gluconeogenic switch in members of the *Sulfolobales*. In order to unravel the regulatory properties, the genes annotated as PK (*SSO0981*), PEPS (*SSO0883*) and putative PEPS or PPDK (*SSO2820*) of *Sul. solfataricus* P2 were cloned, recombinantly expressed, purified and characterized. Here we report the regulatory properties at the level of PEP/pyruvate conversion and compare the findings to other well characterized hyperthermophilic Archaea.

## Materials and Methods

### Cloning and Expression of the PEPS, the PK and the Putative PEPS/PPDK of *Sulfolobus solfataricus* in *Escherichia coli*

The genes encoding the PK (*SSO0981*) and the putative PEPS/PPDK (*SSO2820*) were amplified by PCR mutagenesis using the KOD HiFi DNA Polymerase (Novagen). For the PCR reaction the following primer sets were used (55°C annealing temperature).


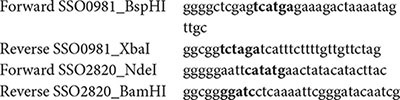


After digestion with *BspHI* and *XbaI* for *SSO0981* as well as *NdeI* and *BamHI* for *SSO2820* the constructs were ligated into pET324 (*SSO0981*) and pET11c (*SSO2820*), respectively. The gene encoding PEPS (*SSO0883*), was synthesized (codon optimized for expression in *Escherichia coli*; for sequence see Supplementary Material) and cloned into pET11c by Eurofins MWG (Ebersberg, Germany).

The resulting constructs (PK_pET324, SSO2820_pET11c, PEPS_pET11c) were transformed into competent *E. coli* Rosetta (DE3) pRIL cells (Novagen). The cells were cultivated in a 5 L fermenter (Minifors, Infors HT) with 4.5 L of LB-medium, 100 μg/ml ampicilin, 34 μg/ml chloramphenicol, and 25 μL/L (v/v) of antifoam 204 (Sigma). The fermenter was inoculated with 2% (v/v) of a pre-culture of the respective expression strain and fermentation was performed at 37°C, 750 rpm and aeration (1 bar). Gene expression was induced at OD_600_ of 0.5–0.8 by adding isopropyl-β-D-thiogalacto-pyranoside to a final concentration of 1 mM. The cells were grown until the stationary phase was reached and cells were harvested by centrifugation (5465 × *g*, 20 min, 4°C) and stored at -80°C (∼10 g wet weight).

### Purification of the Recombinant Proteins

For the purification of PK (SSO0981) and the putative PEPS/PPDK (SSO2820) cells were re-suspended in 30 mL 100 mM HEPES/KOH, pH 7 [room temperature (RT)] [3 mL buffer/g cells (wet weight)]. Cell disruption was performed by sonification (ultrasound processor UP 200s, Hielscher Ultrasonics GmbH) at an intensity of 50% for 4 × 5 min interrupted by intervals of 30 s on ice. Cell debris and unbroken cells were removed by centrifugation (21,000 × *g*, 1 h, 4°C). The resulting crude extract was diluted 1:1 with 100 mM HEPES/KOH, pH 7 (RT). Recombinant *Sul. solfataricus* proteins were enriched via heat precipitation for 20 min at 80°C, followed by centrifugation (21,000 × *g*, 1 h, 4°C). For the PEPS/PPDK the protein fraction was directly used for enzymatic analysis, whereas the PK was further purified.

For ion exchange chromatography the PK protein solution was dialyzed against 20 mM HEPES/KOH (pH 7, RT). Ion chromatography was performed using a continuous bed ion exchange column (12 mL, Q-Sepharose Fast Flow, GE Healthcare). After equilibration of the column the protein sample was applied and proteins were eluted via a continuous gradient of 0–1 M NaCl in 20 mM HEPES/KOH, pH 7 (RT) (336 mL, flow rate: 5 mL/min). Fractions containing the target enzymes were pooled and dialyzed against 5 L of 20 mM HEPES/KOH, pH 7 (RT) for 2 h. The sample volume was reduced to 4.5 mL via centrifugation (3000 × *g*, 4°C) using Vivaspin 20 diafiltration cups (10,000 MWCO PES, Sartorius Stedium Biotech). Gel filtration was performed applying the protein samples to HiLoad 26/60 Superdex^TM^ 200 prep grade (GE Healthcare) pre-equilibrated in running buffer [50 mM HEPES/KOH, 300 mM KCl, pH 7 (RT)]. Protein separation was performed at a flow rate of 2 mL/min. Fractions containing the target protein were pooled and the protein sample was stored at 4°C.

For the purification of PEPS (SSO0883) cells were re-suspended in 100 mM Tris/HCl pH 7 (70°C), 20 mM β-mercaptoethanol and 1 mM MgCl_2_. After sonification [3 × 5 min, (intensity of 60%)] and centrifugation (21,000 × *g*, 30 min, 4°C) the heat precipitation (20 min at 75°C) was performed without further dilution followed by centrifugation (21,000 × *g*, 30 min, 4°C). Due to the instability of the PEPS no further purification steps were performed.

### Enzyme Assays

#### Pyruvate Kinase

The assay was performed continuously at 50, 60, and 65°C in the presence of 100 mM HEPES/KOH (pH 6.5 at the respective temperature), 0.2 mM NADH, 4 U of lactate dehydrogenase (LDH, rabbit muscle; Sigma), 0–5 mM PEP, 5 mM ADP, 0.75–2 mg/mL of protein, and 10 mM MgCl_2_ (total volume of 500 μL). Enzyme activity was determined by monitoring the decrease in absorption at 340 nm. Reactions were started by adding the substrate PEP.

Pyruvate kinase activity at 70°C and 80°C was determined using a discontinuous enzyme assay, performed in 0.1 M HEPES (pH 6.5 at 70 or 80°C) with 0.75–2 μg of protein in presence of 10 mM MgCl_2_ in a total volume of 120 μL. Reactions were started by the addition of PEP and samples were removed after 0, 1, 2, 3, and 4 min and stored on ice. The tested range of substrate and co-substrate concentrations were 0.01 mM - 10 mM for PEP and 0.02 mM -5 mM for ADP. Formed pyruvate was detected in an indicator reaction at 37°C in 0.1 M HEPES/KOH (pH 7, RT) containing 0.5 mM NADH and 4 U LDH (total volume 500 μL, 340 nm).

Assays in presence of effectors were performed in a continuous assay system (500 μL total volume) at 60°C under half-saturating substrate conditions for PK (0.03 mM PEP) in the presence of 2.3 μg protein, 0.2 mM NADH, 4 U LDH (rabbit-muscle, Sigma-Aldrich), 2 mM ADP and 10 mM MgCl_2_. Following effectors were used in final concentrations of 1 mM: fructose 6-phosphate, fructose 1,6-bisphosphate, glucose 6-phosphate, glucose 1-phosphate, trehalose 6-phosphate, fructose 1-phosphate, ribose 1-phosphate, UDP-glucose, 2-keto-3-deoxy-6-phospho-D-gluconate, 3-phosphoglycerate, 2-phosphoglycerate, dihydroxyacetone phosphate, glyceraldehyde 3-phosphate, citrate, malate, oxaloacetate, α-ketoglutarate, succinate, fumarate, 2-oxo-glutamate, isocitrate, KP_i_, PP_i_, AMP, ATP, UTP, CTP. More detailed analyses were performed in presence of ATP and isocitrate by varying the concentrations between 0.1 and 6 mM.

#### Phosphoenolpyruvate Synthetase

Phosphoenolpyruvate synthetase activity in both the anabolic and catalytic direction was determined at 70°C using a discontinuous assay according to Eyzaguirre et al. (1982). The standard assay (total volume 25–50 μL) was performed in 100 mM Tris/HCl, pH 7.0 (at 70°C), in the presence of 30 mM β-mercaptoethanol, 10 mM MgCl_2_ and 20 μg of purified enzyme.

For the anabolic direction, 0.1–10 mM pyruvate and 0.1–10 mM ATP were used as substrate and cosubstrate, respectively. The reaction was stopped at 0, 2, 4, 6, 8, and 10 min by transferring the samples on ice. The formed PEP was determined at RT after 60–300 s in 0.5 mL 100 mM Tris/HCl (pH 7.0), 20 mM MgCl_2_, 1 mM ADP and 0.8 mM NADH by calculating the decrease in absorption at 365 nm (ε_25°C_ = 3,4 mM^-1^ cm^-1^) using 10 U LDH (rabbit muscle) and 5 U PK (rabbit muscle) as auxiliary enzymes. To clarify whether AMP/Pi or ADP is produced in the anabolic direction, the samples obtained at different time points [using 6 mM pyruvate and 10 mM ATP as (co)substrates] were transferred to centrifugal concentrators (VIVASPIN 500, Sartorius) to remove the protein. The concentration of PEP in the obtained flow through was determined as described above. In addition, the assay was performed in absence of ADP or with addition of 1 mM ATP and 5 U myokinase (rabbit muscle) to differentiate between the formation of ADP and AMP, respectively.

For the catabolic direction 6 mM PEP, 10 mM K_2_HPO_4_ and 10 mM ADP or AMP were used as substrates and the formed pyruvate was determined as described for PEP detection, however, in absence of the MgCl_2_, ADP and PK. To analyze the regulatory properties of PEPS, various metabolites (α-ketoglutarate, AMP, ADP, glyceraldehyde-3-phosphate, 3-phosphoglycerate, 2-phosphoglycerate, dihydroxyacetone phosphate, glucose 1-phosphate, glucose 6-phosphate, oxaloacetate, fructose 6-phosphate, fructose 1,6-bisphosphate) were tested for their influence on the catalytic activity of the enzyme at half-saturating concentrations of ATP and pyruvate.

#### Pyruvate:Phosphate Dikinase

For monitoring PPDK activity in the anabolic direction (PEP formation), the discontinuous assay described for PEPS containing 100 mM Tris/HCl, pH 7.0 (55 or 70°C), 20 mM β-mercaptoethanol, 6 mM pyruvate, 15 mM ATP, 10 mM MgCl_2_ and additional 5 mM KP_i_ was used. Pyruvate formation by PPDK (catabolic direction) was determined either at 70°C using a discontinuous assay or at 55°C in a continuous assay. In both cases, standard assays were performed in 100 mM Tris/HCl, pH 7.0 (55 or 70°C) in the presence of 3 mM PEP, 5 mM AMP and 4 mM Mg-EDTA. The reaction was started by addition of 1 mM PP_i_. In the continuous assay, the reaction mixture additionally contained 0.8 mM NADH and 10 U LDH, and pyruvate formation was followed directly by the decrease in absorption at 366 nm [∈55°C = 3.33 mM^-1^ cm^-1^ (Fabry and Hensel, 1987)]. At 70°C a discontinuous assay was used and the amount of pyruvate formed by PPDK after 20–120 s (sample volume 25–50 μL) was determined at RT in 500 μL total volume, 100 mM Tris/HCl (pH 7.0), 0.8 mM NADH using 10 U LDH as auxiliary enzyme.

## Results

Archaea utilize modifications of the classical metabolic pathways which are often characterized by novel enzymes with different regulatory properties. The regulation in dependence of the offered carbon source and the switch between glycolytic and gluconeogenic growth is so far only scarcely addressed. Here we identify and characterized the enzymes involved in PEP-pyruvate conversion in *Sul. solfataricus*.

### Pyruvate Kinase

**Figure 1:**
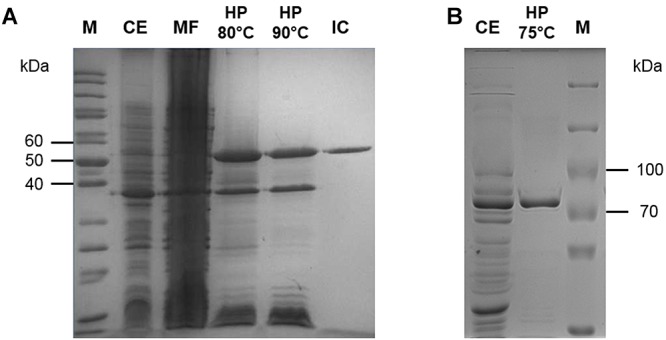
Purification of the PK **(A)** and PEPS **(B)** from *Sul. solfataricus* heterologously expressed in *E. coli*. Protein fractions were separated via SDS-PAGE [12.5% (w/v) polyacrylamide, stained with Coomassie brilliant blue]. M, protein marker [PageRuler Unstained Protein Ladder **(A)**, PageRuler Plus Prestained Protein Ladder **(B)**, Thermo Scientific]; CE (10 μg), crude extract; MF, membrane fraction; HP (5 μg), heat precipitation; IC (2 μg): PK fractions after ion exchange chromatography.

The open reading frame (ORF) SSO0981 is annotated as PK in the *Sul. solfataricus* genome and was cloned into the vector pET324. The enzyme was expressed heterologously in *E. coli* using the pET expression system. The protein was purified by heat precipitation (20 min 90°C) and ion exchange chromatography (elution at 360 mM NaCl). PK exhibits a molecular mass of approximately 50 kDa (Figure 1A), which matches the calculated molecular mass of 49.8 kDa. The total yield of protein was 3 mg out of 9.5 g of cells (wet weight).

**Figure 2:**
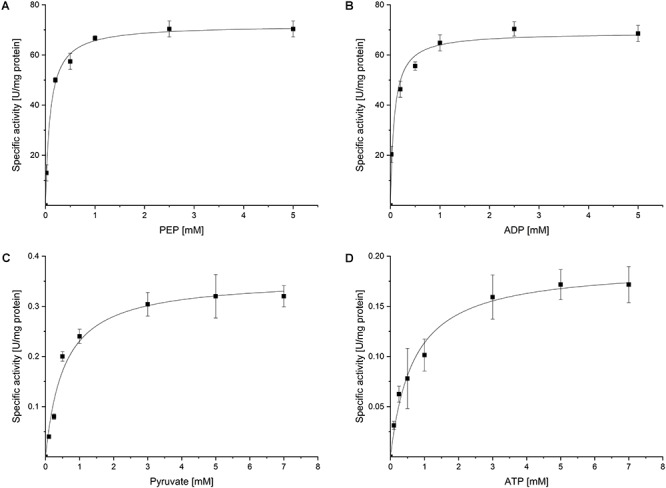
Characterization of PK and PEPS from *Sul. solfataricus* at 70°C. PK activity with PEP **(A)** and ADP **(B)** and PEPS activity with pyruvate **(C)** and ATP **(D)** was determined. Both enzymes follow classical Michaelis–Menten kinetics at 70°C. All measurements were performed in triplicate, the points in the figures are the mean values and the error bars indicate the standard deviation. Kinetic parameters are given in Tables 1, 2.

For the ORF *SSO0981* PK activity could be confirmed and the enzyme was characterized at 50, 65, 70, and 80°C (Table 1). The *Sul. solfataricus* PK follows classical Michaelis–Menten kinetics for PEP (0–6 mM) and ADP (0–6 mM) at the different temperatures tested (Figure 2 and Supplementary Figure 1). Only for PEP concentrations below 1 mM at 80°C a slight deviation was observed. For PEP, the specific activity (*V*_max_) increased with temperature, with the highest specific activity at 80°C with 88.7 U/mg. The determined affinity for PEP (*K*_m_-values) increased from 0.23 mM at 50°C to 0.12 at 65°C and 0.09 mM at 70°C. Only at 80°C a decrease in affinity (*K*_m_-value 0.26 mM) was observed. Therefore, the highest catalytic efficiency was determined at 70°C (628.0 mM^-1^s^-1^) with a 2.2-fold reduction at 80°C (281.0 mM^-1^s^1^). The catalytic efficiency for the co-substrate ADP showed a similar trend with 244.3 mM^-1^s^-1^ at 50°C, 385.8 mM^-1^s^-1^ at 65°C, 851.9 mM^-1^s^-1^ at 70°C and 710.5 mM^-1^s^-1^ at 80°C (Table 1). These observed differences in catalytic efficiency are mainly due to the changes of *K*_m_-values at the different temperatures.

**Table 1:** Kinetic parameters of the *Sul. solfataricus* PK for PEP and ADP determined at 50, 65, 70, and 80°C.

	Temp	*V*_max_	*K*_cat_	*K*_m_	*K*_cat_/*K*_m_
Substrate	[°C]	[U/mg]	[s^-1^]	[mM]	[mM^-1^s^-1^]
PEP	50	47.9	39.7	0.23	137.3
	65	60.4	50.1	0.12	435.8
	70	70.4	58.4	0.09	628.0
	80	88.7	73.0	0.26	281.0
ADP	50	48.3	40.1	0.16	244.3
	65	63.7	52.9	0.14	385.8
	70	70.8	58.8	0.07	851.9
	80	96.7	80.3	0.11	710.5

*For the final enzyme characterization one enzyme preparation was used and the enzyme assays were performed in triplicates. The mean values and standard deviation is shown in Figure 2 and Supplementary Figures 1, 3*.

Numerous metabolites and signaling compounds (fully listed in material and methods) were tested as effectors of *Sul. solfataricus* PK activity at half-saturating concentration of PEP and ADP. No activator was identified, but ATP and isocitrate acted as inhibitors (Supplementary Figure 2). The product inhibition by ATP and isocitrate was studied in more detail: Approximately 0.8 mM of ATP or isocitrate was required for 50% inhibition (50% residual activity) at 60°C. The inhibition could not be reversed by the addition of PEP or ADP. Additionally, well known activators of classical bacterial and eukaryotic PKs, i.e., AMP and FBP were tested as possible effectors, but showed no effect and did not reverse the inhibition of ATP.

### Phosphoenolpyruvate Synthetase (PEPS)

The ORF SSO0883 is annotated as PEPS in the *Sul. solfataricus* genome. The encoding gene was synthesized with codon optimization for expression into *E. coli* using the pET expression system (vector pET11c). The enzyme was partially purified by heat precipitation (20 min at 75°C). PEPS exhibits a molecular mass of 89–92 kDa (Figure 1B), which matches approximately the calculated molecular mass of 89.3 kDa. Due to its instability no further purification steps were applied and aliquots of the enzyme were directly stored at -80°C in the presence of 20% (v/v) glycerol.

The recombinant PEPS from *Sul. solfataricus* (SSO0883) was shown to catalyze the ATP-dependent formation of PEP. PEPS activity was assayed in the anabolic direction following PEP formation in a discontinuous assay at 65, 70, and 80°C. The enzyme follows classical Michaelis–Menten kinetics for pyruvate and ATP (Figure 2 and Supplementary Figure 3). Further analysis revealed that in agreement with the classical PEPS reaction AMP and Pi rather than ADP is formed in the anabolic direction. Kinetic properties could not be determined at 80°C properly, which might be due to the heat instability of PEPS. The *V*_max_-value at 70°C is slightly increased and the *K*_m_-value decreased resulting in a higher catalytic efficiency at 70°C compared to 65°C (0.75 mM^-1^s^-1^ at 65°C and 1.04 mM^-1^s^-1^ at 70°C, respectively, Table 2). In the catabolic direction, PEPS activity was determined as 0.032 U/mg at 70°C using PEP, AMP and P_i_ as substrates, which is 10% of its anabolic activity under comparable assay conditions (data not shown). No enzyme activity was detected in the catabolic direction using ADP as phosphate acceptor.

**Table 2:** Kinetic parameters of *Sul. solfataricus* PEPS for pyruvate and ATP determined at 65 and 70°C, respectively.

	Temp	*V*_max_	*K*_cat_	*K*_m_	K_cat_/K_m_
Substrate	[°C]	[U/mg]	[s^-1^]	[mM]	[mM^-1^s^-1^]
Pyruvate	65	0.27	0.41	0.54	0.75
	70	0.32	0.48	0.46	1.04
ATP	65	0.17	0.25	0.47	0.53
	70	0.22	0.33	0.61	0.53
PEP + AMP	70	0.03	0.05	ND	ND

*For the final enzyme characterization one enzyme preparation was used and the enzyme assays were performed in triplicates. The mean values and standard deviation is shown in Figure 2 and Supplementary Figures 1, 3. ND, not determined*.

As shown in Supplementary Figure 4, PEPS activity was inhibited by α-ketoglutarate and AMP (around 80% residual activity in presence of 1 mM inhibitor). All other metabolites tested (fully listed in Section “Material and Methods”) showed no effect on PEPS activity.

### Phosphoenolpyruvate Synthetase (PEPS) or Pyruvate:Phosphate Dikinase (PPDK)

The predicted PEPS/PPDK (SSO2820, PpsA-2) was cloned into pET11c. The protein was expressed in *E. coli* and purified by heat precipitation (20 min at 80°C) to confirm the respective enzyme activity. For the predicted PPDK (SSO2820) no interconversion of PEP and pyruvate could be detected under different test conditions (PPDK, PK, and PEPS assay), demonstrating that the protein encoded by SSO2820 exhibits neither PPDK, PEPS, nor PK activity (data not shown).

## Discussion

A general feature of all (hyper)thermophilic Archaea analyzed hitherto is the lack of classical bacterial or eukaryotic control points at the beginning and end of the EMP pathway (for review see Bräsen et al., 2014). The archaeal ATP-dependent hexokinase, ADP-dependent glucokinase, ATP-, ADP-, and PP_i_-dependent PFKs lack allosteric properties and also the archaeal PKs typically exhibit reduced, if at all any, regulatory potential. In *Sul. solfataricus* three candidate genes were annotated for PEP-pyruvate conversion, i.e., PK, PEPS and PEPS/PPDK, which were recombinant expressed in *E. coli* and the corresponding proteins were characterized for their enzymatic and regulatory properties.

### Pyruvate Kinase

Pyruvate kinase catalyzes the final step in glycolysis, the conversion of PEP to pyruvate with the concomitant synthesis of ATP via substrate-level phosphorylation (Reynard et al., 1961). For the ORF SSO0981 annotated as PK the respective activity was confirmed. The enzyme like other PKs requires magnesium or other divalent metal ions for activity (Susan-Resiga and Nowak, 2004). However, as reported previously for other archaeal PKs, with the exception of the *Thermoplasma acidophilum* enzyme, the enzyme does not require monovalent cations such as K^+^ or NH_4_^+^ for activity as described for many other PKs from Bacteria and Eukaryotes (Schramm et al., 2000; Oria-Hernández et al., 2005; Bräsen et al., 2014). The characterization of the *Sul. solfataricus* PK at temperatures from 50°C to 80°C revealed highest catalytic efficiency at 70°C (Table 1 and Supplementary Figure 1). No positive cooperativity for PEP or ADP was observed at the different temperatures but effector studies revealed a significant non-competitive inhibition of the *Sul. solfataricus* PK by ATP and isocitrate (*K*_i_ = 0.8 mM). The addition of (co) substrate or activators of classical bacterial and eukaryotic PKs (i.e., AMP and FBP) did not affect the inhibition of the *Sul. solfataricus* PK. Therefore the energy charge of the cell (ATP) as well as the availability of building blocks for biosynthesis in the citric acid cycle (isocitrate) seem to play important roles in the regulation of glycolysis in *Sul. solfataricus.*

To date, only a few PKs from the archaeal domain have been biochemically characterized, i.e., of the hyperthermophiles *Thermoproteus tenax* (Schramm et al., 2000), *Pyrobaculum aerophilum* (Solomons et al., 2013), *Aeropyrum pernix* and *Archaeoglobus fulgidus* (Johnsen et al., 2003) as well as the moderate thermoacidophile *Tpl. acidophilum* (Potter and Fothergill-Gilmore, 1992). For most of the archaeal PKs positive cooperativity toward PEP or ADP or for the *Tpt. tenax* PK toward PEP and Mg^2+^ was reported. In general, archaeal PKs exhibit no allosteric regulation by classical effectors of bacterial and eukaryotic PKs such as fructose 1,6-bisphosphate (F1,6BP), fructose 2,6-bisphosphate (F2,6BP), AMP or other sugar phosphates (Siebers and Schönheit, 2005). Only the *Tpl. acidophilum* PK was shown to be activated by AMP (Potter and Fothergill-Gilmore, 1992). For the PK of *Pyb. aerophilum* 3-phosphoglycerate (3-PG) was identified as unusual activator (Solomons et al., 2013). Inhibition by ATP was so far only reported for the *Arc. fulgidus* PK, which, however, could be reversed by higher PEP and ADP concentrations, suggesting a competitive inhibition (Johnsen et al., 2003). Notably, the PK from the hyperthermophilic bacterium *Thermotoga maritima* exhibits conventional bacterial regulatory properties demonstrating that the differences found for archaeal PKs represent no general adaptation to high temperature (Johnsen et al., 2003).

### PEPS/PPDK

The ORFs SSO2820 and SSO0883 are annotated as PEPS/PPDK and PEPS, respectively. In the lower shunt of the EMP pathway, these enzymes catalyze the interconversion of pyruvate and PEP. PEPS is a member of the PEP-utilizing enzyme family, which also comprises PPDK. PEPS activity was demonstrated for many bacterial and few archaeal species (e.g., Cooper and Kornberg, 1965; Hutchins et al., 2001; Tjaden et al., 2006). Deletion mutant experiments in *Escherichia coli* and *Salmonella typhimurium* revealed that the enzyme is crucial for growth on C3 substrates like pyruvate, lactate and alanine (Cooper and Kornberg, 1967; Smyer and Jeter, 1989). In accordance with this gluconeogenic function, for the archaeon *Methanothermobacter thermoautotrophicus* an essential role for autotrophic growth in the presence of CO_2_ (Eyzaguirre et al., 1982) and for *Pyrococcus furiosus* on pyruvate (Schäfer and Schönheit, 1993) was demonstrated.

**Figure 3:**
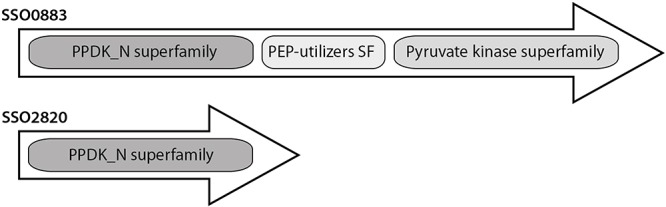
Conserved domain organization of the PEPS and putative PEPS/PPDK from *Sul. solfataricus*.

However, the sequence of SSO2820 does not match the typical size of characterized PEPSs and PPDKs (Figure 3). The protein comprises only the conserved PPDK_N superfamily domain (pfam01326, amino acid location 31–340) involved in PEP/Pyruvate binding. The additional histidine domain (pfam00391, PEP-utilizing enzyme, mobile domain; cl00215, Aconitase swivel domain, amino acid location 353–461) and the nucleotide domain (pfam02896 PEP-utilizing enzyme, TIM barrel domain; cl21481, malate synthase; amino acid location 479–784) characteristic for PEPSs and PPDKs are missing (Herzberg et al., 1996; Lim et al., 2007) (Figure 3).

Both ORFs were heterologously expressed in *E*. *coli* and analyzed. Only the three-domain protein SSO0883 exhibited reversible PEPS activity, while the truncated SSO2820 showed neither PEPS nor PPDK (or PK) activity. Notably, SSO2820 was identified as one of the differentially phosphorylated proteins in a previous phosphoproteome study in *Sul. solfataricus* (Esser et al., 2012; Esser et al., 2016). The protein was phosphorylated under glucose but not under tryptone growth conditions suggesting a so far unknown function in the carbon switch, may be a role in the regulation of PK or PEPS or the PEP-pyruvate conversion.

For the PEPS (SSO0883) gluconeogenic and glycolytic activity with PEP, AMP/P_i_ (PEPS activity) could be detected although the gluconeogenic direction was clearly preferred. The highest catalytic efficiency in the anabolic direction was observed at 70°C (1.04 mM^-1^s^-1^ at 70°C). For the PEPS from *Pyr. furiosus* also reversible PEPS activity with a clear preference for the gluconeogenic direction for PEP formation was reported (Hutchins et al., 2001). Also for the PEPSs from *Mba. thermautotrophicus* and *Tpt. tenax* an *in vivo* anabolic function was confirmed (Eyzaguirre et al., 1982; Tjaden et al., 2006). Like for the *Sul. solfataricus* PEPS (inhibition by AMP and α-ketoglutarate, 80% residual activity in presence of 1 mM inhibitor) also for the *Tpt. tenax* PEPS allosteric regulation [i.e., inhibition by α-ketoglutarate, AMP and ADP (*K*_i_ of 0.6, 0.5 and 2.6 mM, respectively)] was shown. The authors proposed an inhibition by low energy charge of the cell and a linkage of the EMP pathway to amino acid biosynthesis (Tjaden et al., 2006).

Therefore the PEPS (SSO0883) of *Sul. solfataricus* is a unidirectional anabolic enzyme that is inhibited by low energy charge of the cell (i.e., AMP) and α-ketoglutarate, an intermediate of the citric acid cycle. The ORF SSO0883 annotated as PEPS/PPDK is only a truncated protein and possesses neither PEPS or PPDK activity and a possible function in the regulation of PEP-pyruvate conversion is predicted.

### Current Insights Into the Regulation of the Carbon Switch in *Sul. solfataricus*

*Sulfolobus solfataricus* misses a functional PFK and therefore the branched ED pathway with ATP-dependent KDG kinase (spED) and glycerate kinase (npED) is used for glycolysis (Ahmed et al., 2005). In addition, the ATP-dependent hexokinase provides sugar phosphates for the generation of pentoses (reverse ribulose monophosphate pathway), glycogen and trehalose (Nishimasu et al., 2006; Kouril et al., 2013b).

In *Sul. solfataricus* and other (hyper)thermophiles with optimal growth at 80°C a major control site in the EMP/spED pathway was identified at the level of triosephosphate conversion, which has been discussed in respect to metabolic thermoadaptation (Kouril et al., 2013a; Bräsen et al., 2014). Triosephosphates are especially instable at high temperatures [half-lives: GAP 12.4 min at 70°C, DHAP 30.8 min at 70°C, and 1,3-bisphosphoglycerate (BPG) 1.6 min at 60°C (Kouril et al., 2013a)] and for DHAP and GAP the formation of highly toxic methylglyoxal is reported (Gauss et al., 2014). Due to this metabolic burden, the accumulation of thermolabile triosephosphates seem to be critical for (hyper)thermophiles and a sophisticated regulation to avoid their accumulation in the cell is required: (i) Glyceraldehyde 3-phosphate (GAP) conversion is exerted by the solely gluconeogenic enzyme couple GAPDH and PGK and the catabolic, non-phosphorylating NAD(P)^+^-dependent GAPDH (GAPN). GAPN catalyzes the direct oxidation of GAP to 3-phosphoglycerate omitting substrate level phosphorylation and is typically activated by G1P, an intermediate in polymer degradation/glycogen metabolism. The glycolytic activity of the GAPDH is hampered by a tremendously high *K*_m_-value for P_i_ (409 mM) and the PGK, although it prefers the catabolic reaction, is inhibited by low energy charge of the cell [*K*_i_ (ADP) = 1.14 mM] (Kouril et al., 2013a). (ii) The TIM for GAP and DHAP interconversion possesses an anabolic/gluconeogenic function in *Sul. solfataricus* and is inhibited by PEP (*K*_i_ = 0.66 mM) and 3-phosphoglycerate (*K*_i_ = 0.4 mM), which might allow fine-tuning of metabolism by redirecting the carbon flux in the glycolytic direction (Kouril et al., 2013a). (iii) The further conversion of triosephophates in the gluconeogenic direction is catalyzed by a solely anabolic, bifunctional FBP aldolase/FBPase (Say and Fuchs, 2010; Kouril et al., 2013a). (iv) In addition, the inhibition of the glycerate kinase by glycerate (*K*_i_ = 1 mM) was demonstrated and a function as throttle valve in the npED pathway to redirect the carbon flux in the gluconeogenic direction was proposed (Kouril et al., 2013b).

In this study we analyzed PEPS and PK activity in *Sul. solfataricus*: (i) The unidirectional PK is only active in the glycolytic direction with a catalytic efficiency of 628.0 mM^-1^s^-1^ (PEP, 70°C; Table 1) and is allosterically regulated (i.e., via ATP and isocitrate), which is a well known regulatory mechanism for bacterial and eukaryotic PKs but seems to be rather unusual in Archaea. (ii) The PEPS clearly prefers the gluconeogenic direction (10-times higher activity) with a catalytic efficiency of 1.04 mM^-1^s^-1^ (pyruvate, 70°C, Table 2) and is allosterically regulated by AMP and α-ketoglutarate.

The determined gluconeogenic activity of PEPS was significantly lower (0.32 U/mg) than the activity of the glycolytic counterpart PK (70.4 U/mg) both determined at 70°C (Tables 1, 2). However, the inhibition of the *Sul. solfataricus* PK by ATP (50% residual activity at 0.8 mM ATP at 70°C) is in line with the relatively high K_m_ for ATP of the *Sul. solfataricus* PEPS (0.61 mM at 70°C). Therefore, high energy charge of the cell and availability of building blocks (i.e., isocitrate) will slow down the glycolytic reaction of the PK and trigger the gluconeogenic reaction of PEPS. In contrast, low energy charge of the cell (AMP) and the availability of building blocks in the citric acid cycle for amino acid synthesis (α-ketoglutarate, carbon/nitrogen balance) will inhibit PEPS activity.

In addition to PEPS, BLAST analyses revealed the presence of an alternative pathway for PEP formation via PEP-carboxykinase (PCK, SSO2537, EC4.1.1.32) in *Sul. solfataricus*. PCK catalyzes the GTP-dependent conversion of oxaloacetate (citric acid cycle) to PEP and CO_2_. The enzyme of *Tco. kodakarensis* (AB167819) has been characterized and showed significant similarity to the PCK from *Sul. solfataricus* (Fukuda et al., 2004). The PCK of *Sul. solfataricus* was identified as phosphoprotein suggesting a regulation by post-translational modification (Esser et al., 2012). Therefore, beside PEPS a second gluconeogenic enzyme is available in the cell in order to drive the glycolytic switch. The inhibition of the PK by isocitrate, the precursor in the citric acid cycle for the formation of malate and oxaloacetate via the glyoxylate shunt in *Sul. solfataricus* (Uhrigshardt et al., 2002) might thus enhance gluconeogenesis by favoring PEP formation via PCK.

Therefore, in *Sul. solfataricus* the energy charge of the cell seems to play a major regulatory role, which is demonstrated by the inhibition of the anabolic/gluconeogenic enzymes PEPS and PGK by ADP and the inhibition of the catabolic/glycolytic PK by ATP. In addition, the activity of the citric acid cycle and glyoxylate bypass monitored by the availability of intermediates (i.e., isocitrate, α-ketoglutarate) seems to have an additional control function.

### Comparison to the Regulation of the Carbon Switch in Other Studied Archaea

**Figure 4:**
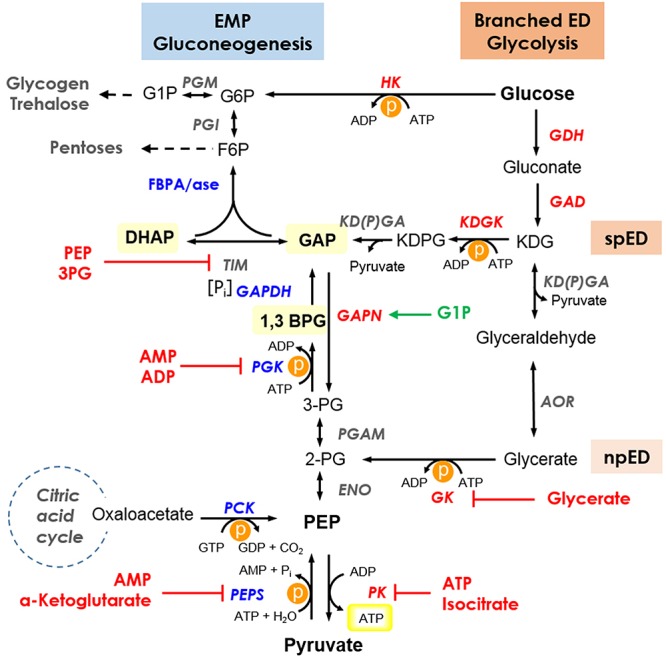
Current insights into the regulation of the branched ED pathway for glycolysis and the EMP pathway for gluconeogenesis in *Sul. solfataricus*. The branching point of the semi-phosphorylative ED (spED) and the non-phosphorylative ED (npED) branch is indicated. Thermolabile intermediates are highlighted and for simplicity, only the cosubstrates for ATP (GTP) converting reactions are shown. The energy consuming reactions are marked by P in an orange circle, the ATP formed by substrate level phosphorylation in the PK reaction is boxed in yellow. Enzymes are shown in italics with reversible, unidirectional/preferred glycolytic and gluconeogenic enzymes in gray, red, and blue, respectively. Inhibitors and activators are shown in red and green, respectively. Enzymes: ENO, enolase; GAD, gluconate dehydratase; GAPDH, glyceraldehyde-3-phosphate dehydrogenase; GAPN, non-phosphorylating GAPDH; GDH, glucose dehydrogenase; GK, glycerate kinase; HK, hexokinase; KDGK, 2-keto-3-deoxygluconate kinase; KD(P)GA, 2-keto-3-deoxy-(6-phospho)gluconate aldolase; PGK, phosphoglycerate kinase; PGAM, phosphoglycerate mutase; PK, pyruvate kinase; PEPS, PEP synthetase; AOR, glyceraldehyde:ferredoxin oxidoreductase; PCK, phosphoenolpyruvate carboxykinase; PGM, phosphoglucomutase/phosphomannomutase; PGI, phosphoglucose/phosphomannose isomerase; FBPA/ase, fructose 1,6-bisphosphate aldolase/phosphatase; TIM, triosephosphate isomerase. Intermediates: G6P, glucose 6-phosphate; G1P, glucose 1-phosphate; F6P, fructose 6-phosphate; GAP, glyceraldehyde 3-phosphate; DHAP, dihydroxyacetone phosphate; 1,3BPG, 1,3-bisphosphoglycerate; 3PG, 3-phosphoglycerate; 2PG, 2-phosphoglycerate; PEP, phosphoenolpyruvate; KDG, 2-keto-3-deoxygluconate; KDPG, 2-keto-3-deoxy-6-phosphogluconate; ATP, adenosine triphosphate; ADP, adenosine diphosphate; AMP, adenosine monophosphate; GTP, guanosine triphosphate; GDP, guanosine diphosphate; P_i_, inorganic phosphate. Pathway: EMP, Embden–Meyerhof–Parnas; ED, Entner–Doudoroff; spED, semi-phosphorylative ED; npED, non-phosphorylative ED.

In contrast to the aerobe *Sul. solfataricus* the obligate anaerobic hyperthermophiles *Pyr. furiosus* and *Tco. kodakarensis* (*Thermococcales*) as well as *Tpt. tenax* use a modified, reversible EMP pathway and only in *Tpt. tenax* the branched ED pathway is active in parallel (Figure 4) (Ahmed et al., 2004; Zaparty et al., 2008). Also in these hyperthermophiles the conserved control point at the level of triosephosphate conversion is established, however, the anaerobes possess in addition to GAPN a ferredoxin dependent GAP:OR (Mukund and Adams, 1995; Reher et al., 2007; Zaparty et al., 2008).

**Figure 5:**
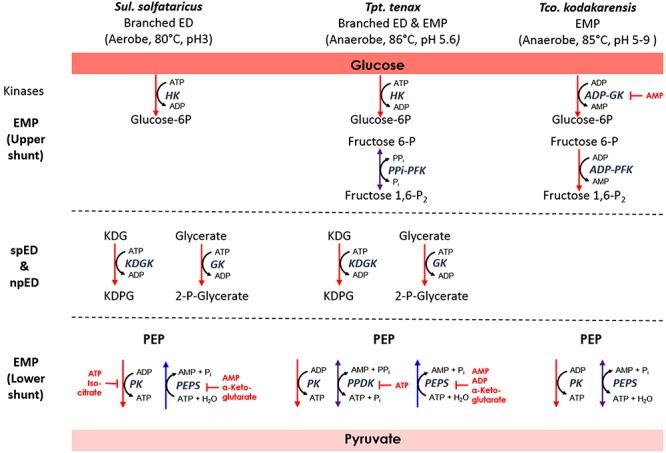
Relationship between sugar phosphorylation and PEP-pyruvate conversion in the archaeal species *Sul. solfataricus, Tpt. tenax*, and *Tco. kodakarensis*. For simplicity, only the cosubstrates for the energy converting reactions in the EMP and branched ED pathway are shown. Enzymes are depicted in italics. Red, blue, or violet arrows indicate unidirectional glycolytic and gluconeogenic as well as reversible reactions, respectively. Established inhibitors are given in red. For abbreviations see Figure 4; PFK, phosphofructokinase; PPDK, pyruvate:phosphate dikinase; fructose 1,6-P_2_, fructose 1,6-bisphosphate; PP_i_, pyrophosphate.

In the different archaeal species there are differences regarding the interconversion of PEP and pyruvate, which seem to be closely linked to the phosphoryl donor in the preparatory phase of glycolysis via sugar kinases (Figure 5 and Table 3).

**Table 3:** Kinetic parameters of enzymes involved in PEP-pyruvate conversion in the archaeal species *Tpt. tenax, Pyr. furiosus* and *Sul. solfataricus*.

		Assay
Organism	Enzyme	Temperature	*V*_max_	*K*_m_	Reference
*Tpt. tenax*	PEPS (anabolic)	70°C	0.45	0.4 (pyruvate)	Tjaden et al., 2006
				1.0 (ATP)	
	PEPS (catabolic)	70°C	Not detectable		
	PPDK (anabolic)	70°C	1.1	0.8 (pyruvate)	
				8.0 (ATP)	
	PPDK (catabolic)	55°C	2.5	0.5 (PEP)	
				0.02 (AMP)	
	PK	50°C	46	0.7 (PEP)	Schramm et al., 2000
*Pyr. furiosus*	PEPS (anabolic)	80°C	14.9	0.11 (pyruvate)	Hutchins et al., 2001
				0.39 (ATP)	
	PEPS (catabolic)	50°C	2.5	0.4 (PEP)	
				1.0 (AMP)	
*Sul. solfataricus*	PEPS (anabolic)	70°C	0.32	0.46 (pyruvate)	This study
				0.61 (ATP)	
	PEPS (catabolic)	70°C	0.03	ND	
	PK	70°C	70.4	0.09	
	PPDK	70°C	Not detectable		

*The respective kinetic parameters for *S. solfataricus* were generated in this study (see Tables 1, 2 and Figure 2) and were retrieved from the literature for *Tpt. tenax* and *Pyr. furiosus* as indicated. ND, not determined*.

#### Thermoproteus tenax

*Thermoproteus tenax* uses three enzymes for PEP/pyruvate conversion: PK, PEPS, and PPDK. PK and PEPS are only unidirectional, whereas the PPDK is bidirectional but more efficient in the glycolytic direction. The PPDK from *Tpt. tenax* is the only archaeal PPDK that has been characterized so far (Tjaden et al., 2006). The enzyme reaction was shown to be reversible with a slight preference for the catabolic direction (2-fold higher catalytic efficiency for pyruvate than for PEP at 70°C). The PPDK of *Tpt. tenax* was independent from monovalent cations and allosteric inhibition by ATP (K_i_ of 0.075 mM) was reported. The *Tpt. tenax* PPDK has been discussed as standby enzyme, which allows for fine tuning of the energy level (Figure 5 and Table 3) (Schramm et al., 2000; Tjaden et al., 2006).

Intriguingly the organism also relies on a reversible PP_i_-dependent PFK for F6P and fructose 1,6-bisphosphate conversion (Siebers et al., 1998). Thus both enzymes, the PP_i_-dependent PFK as well as the PPDK, are reversible enzymes that rely on PP_i_ for the glycolytic and P_i_ for the gluconeogenic direction. In addition, a homolog for a vacuolar-type H^+^-translocating pyrophosphatase from *Pyb. aerophilum* was identified in *Tpt. tenax* enabling generation of a proton motive force (e.g., for ATP synthesis) from PP_i_ (Drozdowicz et al., 1999; Siebers et al., 2011). PP_i_ is often regarded as waste product that has to be removed by cytoplasmic pyrophosphatases in order to drive anabolic processes such as DNA synthesis, however, PP_i_ and polyphosphates in general possess manifold functions and are regarded as ancient energy source (Kornberg, 1995). Therefore, in addition to the energy charge of the cell (ATP/ADP and AMP ratio), that regulates PEPS and PPDK, the PP_i_/P_i_ ratio in the cell seems to be another important control point for the glycolytic switch in *Tpt. tenax*.

#### Thermococcus kodakarensis

The level of PEP-pyruvate conversion as a control point is also well established in *Tco. kodakarensis* (Imanaka et al., 2006). In *Tco. kodakarensis* and *Pyr. furiosus* a functional PK and a bidirectional PEPS were described (Hutchins et al., 2001; Imanaka et al., 2006) (Table 3). Notably, a genetic analysis (*pps* deletion strain) in *Tco. kodakarensis* showed that the PEPS in contrast to PK is indispensable for glycolysis and has only an additional, although not essential function for gluconeogenesis. The *pps* deletion strain displayed no growth on maltooligosaccharides and reduced growth on pyruvate (Imanaka et al., 2006). The sugar phosphorylation in the preparatory part of the EMP pahtway in *Tco. kodakarensis* and *Pyr. furiosus* is catalyzed by unusal ADP-dependent glucokinases and PFKs. Therefore, it was discussed that the ADP will be consumed by the sugar kinases, which will compete with the PK for ADP, and thus PK activity would be lowered and the glycolytic flux reduced (Imanaka et al., 2006). In contrast to PK, PEPS utilizes AMP formed via the ADP-dependent sugar kinase, thus accelerating carbon flux. Thus at high energy/ADP charge of the cell a possible function of PK in conjunction with adenylate kinase (AMP + ATP ↔ 2 ADP) as valve to maintain the intracellular ADP concentration was proposed (Imanaka et al., 2006). In addition, the competitive inhibition of the ADP-dependent glucokinase from *Pyr. furiosus* by AMP (approximately *K*_i_ value 0.06 mM) was reported (Verhees et al., 2002). Therefore the utilization of ADP in the sugar kinase reactions in conjunction with the catabolic PEPS reaction in *Thermococcales*, which reutilizes the formed AMP, seems to represent an important control mechanims to enable an increased glycolytic flux.

## Conclusion

The detailed study of the regulation of the lower shunt of glycolysis in *Sul. solfataricus* in comparison to other archaea reveals different strategies for regulation at the level of PEP-pyruvate conversion. However, as well known for bacteria and eukaryotes it seems to be an important regulation point also in archaeal metabolism. Notably, in the Archaea studied so far there seems to be a close link between the sugar kinases and their phosphoryl donors used in the preparatory phase and the utilized enzymes with different regulatory properties at the level of PEP-pyruvate conversion. These different regulatory strategies might be seen as a sophisticated coordination of energy consuming and generating reactions in glycolysis and gluconeogenesis in order to optimize the direction of carbon flux and energy yield. The observed diversity and presence of modified pathways with different, unusual enzymes is in line with an autotrophic origin of life and the acquirement of metabolic enzymes via massive horizontal gene transfer by Archaea (Nelson-Sathi et al., 2015). These modified archaeal pathways with unusual enzymes often from different enzyme families required novel regulatory mechanisms that are adapted to the respective enzyme repertoire and metabolic challenges such as instability of metabolites at high temperature (Bräsen et al., 2014).

## Author Contributions

PH, BT, LS, and TK performed the experiments. TK and LS wrote the manuscript, which was edited by CB and BS. CB and BS conceived the study. All authors approved the final manuscript.

## Conflict of Interest Statement

The authors declare that the research was conducted in the absence of any commercial or financial relationships that could be construed as a potential conflict of interest.

## Footnotes

1 http://equilibrator.weizmann.ac.il/

## Abbreviations

1,3BPG: 1,3-bisphosphoglycerate
2PG: 2-phoshoglycerate
3-PG: 3-phohoglycerate
ADP: adenosine diphosphate
AMP: adenosine monophosphate
AOR: glyceraldehyde:ferredoxin oxidoreductase
ATP: adenosine triphosphate
CTP: cytidine triphosphate
DHAP: Dihydroxyacetone phosphate
ED: Entner–Doudoroff
EMP: Embden–Meyerhof–Parnas
ENO: Enolase
F1,6BP: Fructose 1,6-bisphophate
F2,6BP: Fructose 2,6-bisphophate
F6P: fructose 6-phosphate
FBPA/ase: fructose 1,6-bisphosphate aldolase/phosphatase
Fructose 1,6-P_2_: fructose 1,6-bisphosphate
G1P: glucose 1-phosphate
G6P: glucose 6-phosphate
GAD: gluconate dehydratase
GAP: glyceraldehyde 3-phosphate
GAPDH: glyceraldehyde-3-phosphate dehydrogenase
GAPN: non-phosphorylating GAPDH
GDH: glucose dehydrogenase
GDP: guanosine diphosphate
GK: glycerate kinase
GTP: guanosine triphosphate
HK: hexokinase
KD(P)GA: 2-keto-3-deoxy-(6-phospho)gluconate aldolase
KDG: 2-keto-3-deoxygluconate
KDGK: 2-keto-3-deoxygluconate kinase
KDPG: 2-keto-3-deoxy-6-phoshogluconate
LDH: lactate dehydrogenase
npED: non-phosphorylative ED
ORF: open reading frame
PCK: PEP-carboxykinase
PEP: phosphoenolpyruvate
PEPS: phosphoenolpyruvate synthetase
PFK: Phosphofructokinase
PGAM: phosphoglycerate mutase
PGI: phosphoglucose/phosphomannose isomerase
PGK: phosphoglycerate kinase
PGM: phosphoglucomutase/phosphomannomutase
P_i_: inorganic phophate
PK: pyruvate kinase
PPDK: pyruvate:phosphate dikinase
PP_i_: pyrophosphate
RT: room temperature
spED: semi-phosphorylative ED
TIM: triosephosphate isomerase
UTP: uridine triphosphate
