# Supplementary material for: The Carbon Switch at the Level of Pyruvate and Phosphoenolpyruvate in Sulfolobus solfataricus P2
Source: Front Microbiol. 2019 Apr 12;10:757. doi: 10.3389/fmicb.2019.00757 (PMC6474364; doi:10.3389/fmicb.2019.00757)
Supplement: Supplementary file 1 [file Data_Sheet_1.pdf]

Supplementary Material

The carbon switch at the level of pyruvate and phosphoenolpyruvate in *Sulfolobus solfataricus* P2

Patrick Haferkamp, Britta Tjaden, Lu Shen, Christopher Bräsen, Theresa Kouril, and Bettina Siebers*

*** Correspondence:**
Prof. Dr. Bettina Siebers
bettina.siebers@uni-due.de

1. Supplementary Data

Gene sequence of the codon optimized PEPS (SSO0883) for expression in *Escherichia coli*:

```
CATATGGGCTCATTTCTCCTGGTTGAAGCGATTAGCTCCGAGGAAGACCTTATCCTCGATGTGTCTCA
AGTAACCAAAGACATGGTGCAGCTCGCGGGTGGTAAAGGGGCCAATCTGGGAGAACTGACTAGCATTG
GAGTGCGTGTTCCTCCGGCATTTATTCTTACCAGCAAAGCCTTCAAATACTTTCTGGAGTACAACAAT
CTCTTCGACAAAATTCGCGATACCTTGAGTTCGAGTGAGACCAGCGAAGAAGCGAGTGAGAAAATTAA
ACAGCTGATCAAAAACGCCAAAATGCCGGATAAACTGTCGTCCATGATCTATCAGGCCTACGATGAAC
TGAGCAAAAAAGTAGGCAAAGAAATCCTGGTAGCGGTACGGTCTTCGGCTACAGCGGAGGACATCGAA
ACAGCGAGTTTTGCTGGTCAGCAGGATACGTACCTGAATGTGACCAAAGATGAGCTGATTGATCGCAT
TAAAGACGTGTGGGCCAGCTTGTACAATGCCCGTGCCATGGAATATCGCAAAAGTAAAGGCATTGACG
ATTTATCGATTCTGATTGCCGTGGTCGTCCAGAAAATGGTTAATTCTCGTTCCGCAGGCGTTATGTTT
ACTCTGCATCCGGTAACGGGTGATGAGAAATACATTATGATCGAATCGAACTGGGGTCTTGGCGAATC
CGTCGTCGGTGGCAAAGTCACGCCTGATGAAGTGCTGATTGAAAAGTCGACCTTACGCATCGTTGAGA
AGAAAGTTTCGAACAAGAACATCAAAATCGTTTACGACAAACAGCTGAAGAAGAATGTCACCATTACC
CTGGACGAGAAAGAAAGTCGCCTGATGAGCATTACGGACGAAGAAGCGATTGAATTAGCAAAACTGGC
TCTGAAAATTGAAGAACACTATAAACGCCCAATGGATATTGAGTGGGCCATTGACAACGATTTGTCCT
TTCCCGAAAACATCTTCATTGTACAGGCACGTCCGGAAACCTTCTGGTCTTCCAAACGCAAAGAAAAC
AAGAATATTGCGGAGAAAAGCGCCCCAATTGGTGGTAAAGTCCTGGTTCGTGGTCTTGCTGCGTCACC
AGGGATTGCCTTTGGCAAAGCAAAGATTATTTTGGATATCAAAGATCCGAAGGTTCATGAGTTCCAGA
AGGGTGAAATTCTGGTGACCAAAATGACTGATCCGGACTGGGTACCGCTCATGAAAATCGCAGGCGCG
ATCATCACCGATGAAGGAGGGATGACGTCACATGCGGCCATTGTCTCACGTGAACTGGGTATCCCCGC
GATCGTTGGTTCTCGCGAAGCAACTAAAATCATCCGCGATAACCAGGAAATCACAGTGGATGCCATCC
GTGGCATTGTGTATGAAGGCAAGGTTCTTCAAACGAGCGAAACAGTGAGCCAACAGGCTCAACCGAGC
ATTGGCATCCAGGGCATTAGTCGCGAGGTGTTGTTAAGCTTATACCCGGTTACCGCGACTAAAATCTA
CATGAACCTGGGCGAGCCAGATGTGATCGATAAATACCTGGACCTGCCGTTTGATGGCATTGGTTTAA
TGCGTATCGAATTTATTGTATCCGAGTGGGTACGCTATCACCCGCTGTATCTGATCAAGATCGGTAAC
GCGGAACTGTTTGTGGACAAACTGGCCGAAGGTATCGCTAAGGTCGCATCTGCGATCTATCCGCGTCC
CGTTGTTGTGCGGTTCAGCGACTTTAAGACGAACGAATACAAAAAACTCATTGGAGGAGAAGAATTTG
AACCTGATGAACGTAACCCGATGATCGGTTGGCGTGGGGTCAGCCGTTATGTGTCGAAAGAATATGAA
CCGGCGTTTCGGTTAGAAGCCAAGGCGATTCGCAAAGTTCGCGAAGAAATGGGCTTAAAGAATGTCTG
GGTTATGTTCCCGTTTGTGCGCACCACCTGGGAACTGGAGAAAGCGATCAAAATCATGGAGGAAGAAG
GCCTGCGCCGCGATTCAGATTTCAAAGTCTGGATTATGGCAGAGGTACCTTCTGTCGTGGTTCTGGCC
GAGGAATTCGCTAAAATTGTGGATGGGTTCTCGGTTGGCTCAAATGATCTCGCACAATTGACGTTGGG
CGTTGATCGCGATTCTGAGTTGCTGGCACGCATGGGCTATTATGATGAGCGCGACCCAGCAGTCCTGG
AAAGCATTCGTAAACTGATTAAAGCGGCGCACAAATATGGGAAAACGGTGAGTATTTGCGGCCAAGCT
CCGAGCGTGTATCCTGCAGTAGTGGAGTATCTTGTCAAAGCTGGTATTGACAGTATTTCAGTGAATCC
CGACGCGGTGATTAACGTGCGCCGTCAAGTGGCTTCCATTGAACAGCAGATCATTCTTCGGAATCTGA
ATGGGAAACGTAAGAACAAATAAGGATCC
```

2. Supplementary Figures and Tables

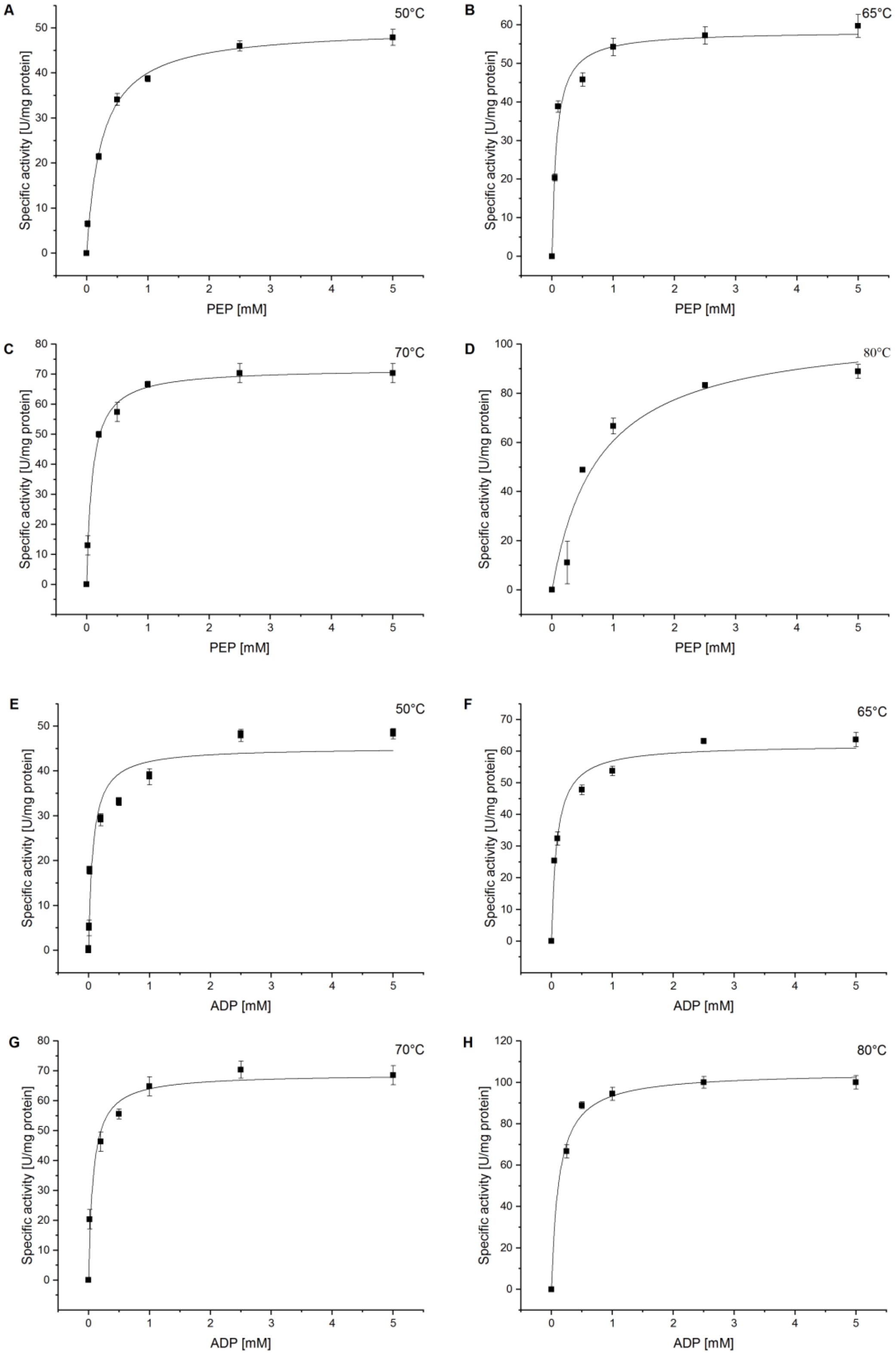

Supplementary Figure 1. Characterization of PK from *Sul. solfataricus*. PK activity with PEP (A-D) and ADP (E-H) was determined at 50°C, 65°C, 70°C and 80°C. All measurements were performed in triplicate, the points in the figures are the mean values and the error bars indicate the standard deviation.

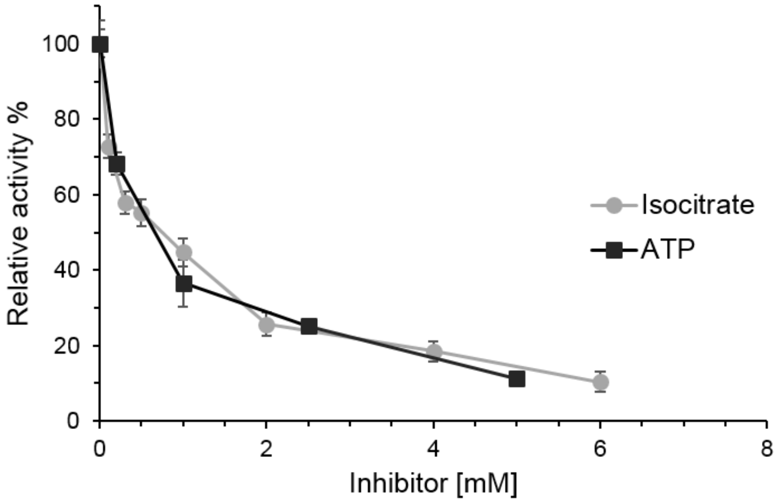

Supplementary Figure 2. Regulation of PK activity. The inhibition of PK activity by ATP and isocitrate was determined at half-saturating concentration of ADP and PEP at 60°C. All measurements were performed in triplicate, the points in the figures are the mean values and the error bars indicate the standard deviation.

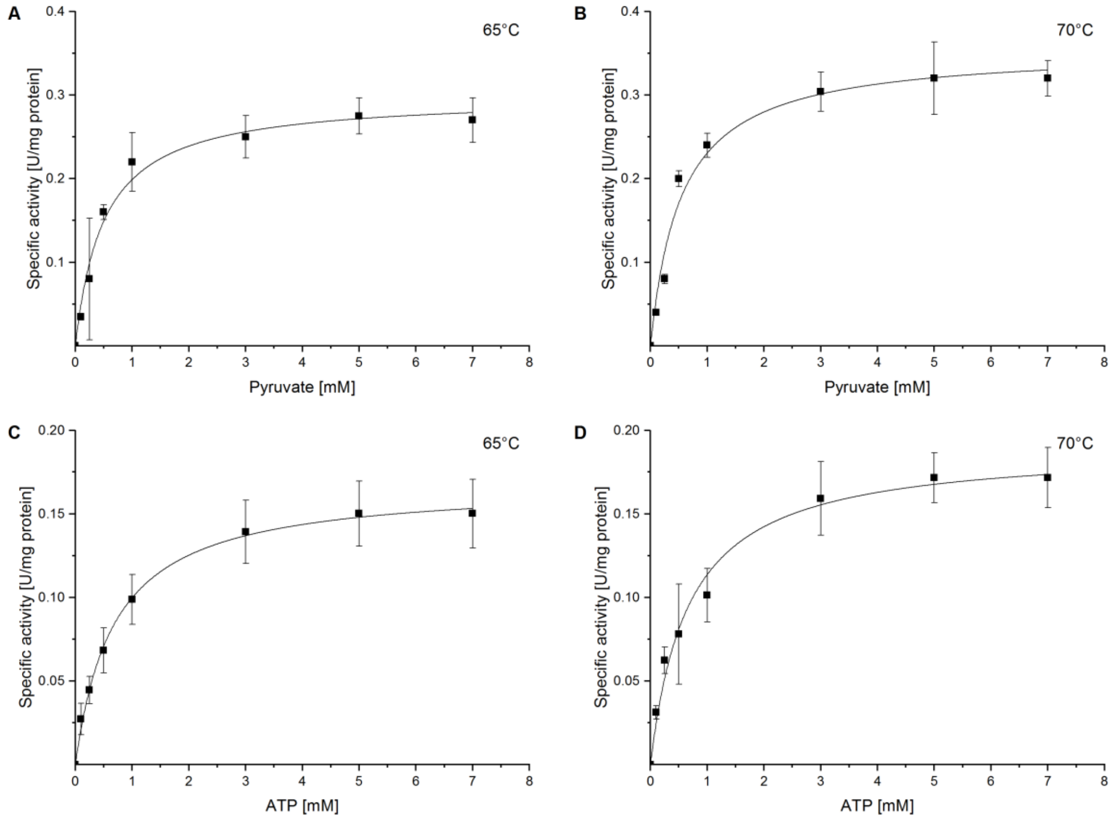

Supplementary Figure 3. Characterization of PEPS from *Sul. Solfataricus*. PEPS activity with pyruvate (A and B) and ATP (C and D) was determined at 65°C and 70°C. All measurements were performed in triplicate, the points in the figures are the mean values and the error bars indicate the standard deviation.

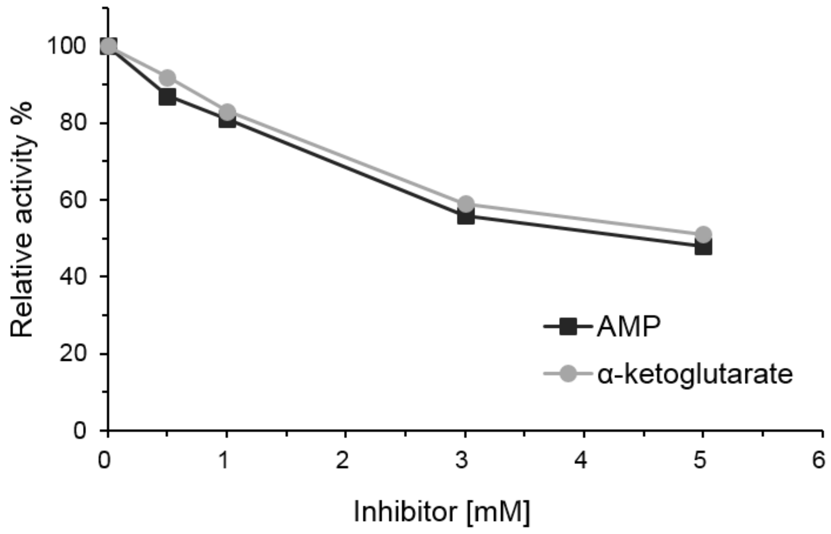

Supplementary Figure 4. Regulation of PEPS activity. The inhibition of PEPS activity by AMP and α-ketoglutarate was determined at half-saturating concentration of ATP and pyruvate at 70°C.
